# Age-Related Mitochondrial Impairment and Renal Injury Is Ameliorated by Sulforaphane via Activation of Transcription Factor NRF2

**DOI:** 10.3390/antiox11010156

**Published:** 2022-01-14

**Authors:** Razia Sultana Mohammad, Mustafa F. Lokhandwala, Anees A. Banday

**Affiliations:** 1 Department of Pharmacological and Pharmaceutical Sciences, College of Pharmacy, University of Houston, Houston, TX 77204, USA; rsmoham2@central.uh.edu (R.S.M.); phar23@central.uh.edu (M.F.L.); 2 Heart and Kidney Institute, College of Pharmacy, University of Houston, Houston, TX 77204, USA

**Keywords:** aging, NRF2, mitochondria, kidney disease, oxidative stress

## Abstract

Age is one of the major risk factors for the development of chronic pathologies, including kidney diseases. Oxidative stress and mitochondrial dysfunction play a pathogenic role in aging kidney disease. Transcription factor NRF2, a master regulator of redox homeostasis, is altered during aging, but the exact implications of altered NRF2 signaling on age-related renal mitochondrial impairment are not yet clear. Herein, we investigated the role of sulforaphane, a well-known NRF2 activator, on age-related mitochondrial and kidney dysfunction. Young (2–4 month) and aged (20–24 month) male Fischer 344 rats were treated with sulforaphane (15 mg/kg body wt/day) in drinking water for four weeks. We observed significant impairment in renal cortical mitochondrial function along with perturbed redox homeostasis, decreased kidney function and marked impairment in NRF2 signaling in aged Fischer 344 rats. Sulforaphane significantly improved mitochondrial function and ameliorated kidney injury by increasing cortical NRF2 expression and activity and decreasing protein expression of KEAP1, an NRF2 repressor. Sulforaphane treatment did not affect the renal NRF2 expression or activity and mitochondrial function in young rats. Taken together, our results provide novel insights into the protective role of the NRF2 pathway in kidneys during aging and highlight the therapeutic potential of sulforaphane in mitigating kidney dysfunction in elders.

## 1. Introduction

Advancing age is a major contributing factor for the development of numerous chronic diseases, and kidneys are one of the vital organs affected, both structurally and functionally, by aging [1,2,3]. Comorbid conditions such as diabetes and hypertension can further exaggerate renal dysfunction and increase morbidity and mortality in elders [2,4]. The mechanisms underlying age-related kidney disease are complex and remain largely unclear. Studies in patients with chronic kidney disease and in animal models of acute and chronic kidney disease showed the pathogenic role of elevated oxidative stress [5,6,7,8]. The prevalent oxidative stress triggers cellular damage causing mitochondrial dysfunction, cytotoxicity, and cell death [9]. Due to high abundance of active transmembrane transporters, kidneys rely heavily on ATP production by the mitochondria, hence they are highly susceptible to any perturbation in mitochondrial function. Together, mitochondrial dysfunction and oxidative stress play a vital role in the pathophysiology of age-related kidney disease [10,11]. Currently, there is no treatment targeting dysfunctional mitochondria in the aging kidneys. A kidney transplant is the only effective strategy available to treat chronic kidney disease, which is very expensive. Therapies targeting oxidative stress lack clinical promise, and mitochondria-targeted therapies have limited efficacy in treating age-related diseases [12,13,14,15,16,17]. Hence, there is an urgent need to understand the underlying molecular mechanisms of age-related kidney damage to provide comprehensive medical care to the elderly. Therefore, identifying the mechanisms regulating mitochondrial dysfunction and oxidative stress could be effective in treating age-related kidney diseases.

The disorganized redox homeostasis in aging kidneys is triggered, in part, by an imbalance in oxidative stress and ROS scavenging antioxidant defense mechanisms [8]. Nuclear factor erythroid 2–related factor 2 (NRF2), a transcription factor belonging to the Cap ‘n’ Collar basic leucine zipper (CNC-bZip) family, is well recognized as the master regulator of cellular antioxidant defense mechanisms [18]. Under basal conditions, NRF2 is bound to KEAP1, a repressor, which leads to ubiquitination and proteasomal degradation of NRF2 [19]. However, during oxidative stress or in the presence of electrophiles, KEAP1-NRF2 binding is disrupted, causing nuclear translocation of NRF2 [20]. In the nucleus, NRF2 dimerizes with sMafs and binds to ARE, a cis-acting enhancer sequence (TCAG/CXXXGC) of the promoters of around 250 genes involved in multiple cellular processes [18,19,21]. Several studies highlighted altered NRF2 transcriptional activity in both older humans and experimental aging models, indicating that NRF2 is important for kidney disease prevention [22]. Experimental models of chronic kidney disease such as diabetic nephropathy, subtotal nephrectomy, and unilateral ureteral obstruction showed decreased NRF2 nuclear translocation along with increased KEAP1 levels [19]. The reversal of age-related loss of DNA binding ability of NRF2 by α-lipoic acid indicates that impairment in NRF2 activity is correctable [23]. Sulforaphane, a sulfur-rich natural compound present in cruciferous vegetables, acts as an indirect antioxidant by inducing glutathione through activation of the NRF2 pathway [24,25]. Previous reports documented NRF2-mediated renoprotective effects of sulforaphane in diabetic nephropathy, ischemic reperfusion injury, and chronic renal allograft dysfunction models [26,27,28]. It is also reported to decrease oxidative stress and inflammation in the rodent models of aging and prevent age-related cardiac and muscular dysfunction [29,30]. The small structure and absolute bioavailability of around 80% makes sulforaphane a promising and translatable compound for NRF2 activation when compared to other polyphenolic NRF2 activators such as resveratrol, curcumin etc. [24].

It is of note that NRF2 regulates essential mitochondrial genes involved in mitochondrial biogenesis, oxidative phosphorylation, and mitophagy [31,32,33,34,35]. NRF2 also plays a role in mitochondrial retrograde trafficking and controls substrate bioavailability during oxidative phosphorylation [36,37]. The beneficial effect of NRF2 activation has been studied in disease models of oxidative stress as well as chemical-induced aging [10,11,38,39,40,41]. However, there is no study reported exploring the mechanistic link between the NRF2 pathway and mitochondrial dysfunction in age-related kidney disease. 

Therefore, the objective of the present study is to investigate the effect of NRF2 activation by sulforaphane on the amelioration of mitochondrial and kidney dysfunction during aging. We hypothesized that sulforaphane could improve mitochondrial and renal function in aged F344 rats. Our results show that sulforaphane remarkably improves NRF2 signaling, increases mitochondrial respiration, and ameliorates renal injury in aged rats.

## 2. Materials and Methods

### 2.1. Animal Studies

Animal studies were performed according to the protocols approved by the Institutional Animal Care and Use Committee (IACUC), University of Houston, Texas. Male Fisher 344 (F344) rats, young (2–4 months old) and aged (20–24 months old), were procured from the National Institute of Aging, Charles river laboratories. Animals were housed in plastic cages, with 12 h light/dark cycle, in the animal care facility of the University of Houston, and acclimatized for one week before any treatment. Animals were divided into four groups after acclimation; (1) Young control, young rats kept on tap water, (2) Aged control, aged rats kept on tap water, (3) Young+Sulforaphane, young rats administered with NRF2 activator, sulforaphane, at a dose of 15mg/kg body weight/day in drinking water, and (4) Aged+Sulforaphane, aged rats administered with sulforaphane (15 mg/kg body weight/day). Sulforaphane dose was chosen based on previous study in a rat model of oxidative stress-induced kidney damage [42]. After four weeks of treatment, fasting blood glucose was measured and the rats were placed in individual metabolic cages for 24 h to collect urine.

#### 2.1.1. Blood Pressure Measurement

At the end of 4 weeks treatment, after collecting urine, blood pressure of the animals was measured according to our published method [43]. Briefly, rats were anesthetized with 4% isoflurane in an induction chamber and anesthesia was maintained with 1.5% isoflurane throughout the surgical procedure using SomnoSuite^®^ anesthesia system (Kent Scientific Corp, Torrington, CT, USA). Blood pressure was measured by catheterizing the left carotid artery by a pressure catheter transducer (Model: SPR-671, Millar Mikro-Tip, Houston, TX, USA) connected to PCU-2000 pressure control unit. Blood pressure was recorded by PowerLab acquisition system and Labchart Pro software program (ADInstruments, Colorado Springs, CO, USA) for one hour, after an initial 30 min period of stabilization. 

#### 2.1.2. Sample Collection

After recording the blood pressure, urine from the bladder and blood from the carotid artery were collected. Plasma was collected by centrifuging blood samples at 3600× *g* for 10 min at 4 °C. Plasma (for creatinine measurement) and bladder urine (for total antioxidant capacity measurement) were flash frozen using liquid nitrogen and stored at −80 °C. The animals were perfused with Krebs–Henseleit Buffer (KHB) (118 mM NaCl, 4.7 mM KCl, 1.2 mM MgSO_4_, 1.25 mM CaCl_2_, 1.2 mM KH_2_PO_4_, 25 mM NaHCO_3_, 11 mM glucose, pH 7.5) maintained at 37 °C. A transverse incision below the rib cage was made and the diaphragm was slightly cut to locate the heart. A cannula was inserted in the left ventricle of the heart and the animals was perfused. Kidneys were removed, blotted dry and weighed. The left kidney was fixed in ice-cold 4% paraformaldehyde for histopathology and immunohistochemistry. The outer capsule of the right kidney was carefully removed and cortical was separated, flash frozen in liquid nitrogen and stored at −80 °C for downstream analysis. 

#### 2.1.3. Measurement of Oxidative Stress

Urinary 8-isoprostane levels were measured by an ELISA kit (Cat# 516351, Cayman Chemicals, Ann Arbor, MI, USA). Briefly, 24 h urine samples were diluted to 1:100 with deionized water and 50 µL of diluted urine was used per well along with suggested kit components as per the manufacturer’s instructions. 

#### 2.1.4. Measurement of Total Antioxidant Capacity

Total antioxidant capacity in bladder urine was measured by a commercially available kit (Cat# 70900, Cayman Chemicals, Ann Arbor, MI, USA) according to the manufacturer’s instructions.

#### 2.1.5. Measurement of Catalase Activity

Catalase activity in the renal cortical tissue homogenates was determined by the method published by Li and Schellhorn with slight modifications [44]. Briefly, the cortical tissue was homogenized in 0.05 M phosphate buffer and centrifuged at 11,000× *g* for 5 min at 4 °C. The supernatant was collected, and protein concentration was estimated by Bradford reagent (Biorad, Hercules, CA, USA). Homogenates were diluted to 1:100 with 0.05 M phosphate buffer and 20 µL of diluted samples in triplicates was added to the wells of Nunc^®^ UV microplate (Cat# 21377 832, Fisher Scientific, Hampton, NH, USA) containing 100 µL of 0.05 M phosphate buffer. The reaction was initiated by adding 25 µL of freshly prepared 30 mM H_2_O_2_. The decomposition of H_2_O_2_ into water and molecular oxygen was monitored at 240 nm for 5 min at 30 s intervals. Catalase activity was reported as the rate of change in absorbance at 240 nm per min per µg of protein. 

#### 2.1.6. Measurement of Plasma Creatinine

Plasma creatinine was measured according to Jaffe’s method using alkaline picrate [45]. Briefly, 12.5 mL of 0.13% picric acid was mixed with 2.5 mL of 1 M NaOH to form an alkaline picrate reagent. Twenty microliters of undiluted plasma was mixed with 40 µL of reagent and absorbance was measured at 490 nm. Samples were analyzed in triplicates and the concentration in plasma was calculated using creatinine standards.

#### 2.1.7. Measurement of Renal Injury Markers

The urine sample, collected for 24 h, were diluted 1:10 with deionized water and protein concentration was measured by Bradford reagent (Biorad) in a 96-well plate at 595 nm using bovine serum albumin as a standard. Albuminuria was measured by EIA. Briefly, clear 96-well plates (R&D Systems, Minneapolis, MN, USA) were coated with 100 µL of capture antibody (sheep anti-rat albumin antibody, Cat# A110-134A, Bethyl Labs, Montgomery, TX, USA) diluted 1:100 with coating buffer (0.05 M carbonate-bicarbonate, pH 9.6, Cat# E107, Bethyl Labs). After 1 h of incubation at room temperature, the capture antibody was removed, plates were washed 3 times with 0.05% Tween 20-Tris (TBST) wash buffer, pH 8.0. Thereafter, 1% BSA blocking buffer was added to the wells and incubated for 30 min at room temperature. Rat serum albumin (Cat# RS-25AL, ICL Labs, Portland, OR, USA) was used as reference standard (range 500 ng/mL–7.8 ng/mL). Urine samples were diluted 1:2000 with the diluent (1% BSA–0.05% TBST). Standards and samples (100 µL) were added to the plates in duplicate and incubated for 1 h at room temp. After 5 washes, the plate was incubated with 100 µL of HRP detection antibody (1:40,000, Cat# A110-134P, Bethyl Labs) for 1 h. Thereafter, the plate was washed and enzyme substrate reaction was carried out using the substrate, color reagent A (Cat# 895000) and color reagent B (Cat# 895001, R&D Systems), for 5–30 min in dark, until light blue color developed and the reaction was stopped using 100 µL of stop solution (Cat# 895926, R&D Systems). The absorbance was measured at 450 nm and albuminuria was calculated. 

Levels of KIM-1, a tubular injury marker, was measured in 24 h urine diluted 1:10 with reagent diluent, using Duoset Rat KIM-1 EIA kit (Cat# DY3689, R&D Systems) according to the manufacturer’s instructions. The absorbance was measured at 450 nm and urinary KIM-1 levels were calculated and reported as pg/24 h.

#### 2.1.8. Measurement of Glomerular Sclerotic Index

Kidneys fixed in ice-cold 4% paraformaldehyde solution were processed in an automated TP1020 tissue processor (Leica Biosystems, Wetzlar, Germany) using series of graded alcohols (Histoprep Reagent Alcohol, Cat# HC6001GAL, Fisher Scientific), SafeClear II Xylene substitute (#23044192) and molten paraffin (#4005, Tissue-Tek V.I.P processing/embedding medium, Electron Microscopy Sciences, Hatfield, PA, USA). The tissue was embedded in paraffin and 5 µm kidney sections were prepared using microtome.

For histopathological assessment of renal morphology, the sections were stained with periodic acid and Schiff’s base using PAS stain kit (# 1016460001, Sigma Aldrich, St. Louis, MO, USA) according to the manufacturer’s instructions. The glomerular damage was assessed by calculating the glomerular sclerotic index (GSI) in a semi-quantitative method [46]. The analysis was done by the observer blinded to treatment groups.

#### 2.1.9. Measurement of Renal Fibrosis Markers

Five-micron kidney sections were deparaffinized, hydrated and subjected to antigen retrieval with 0.01 M sodium citrate buffer pH 6.0, endogenous peroxide quenching with H_2_O_2_. The sections were processed for immunohistochemical staining of renal fibrosis markers using VECTASTAIN IHC kit (Cat# PK-6100, Vector Labs, Burlingame, CA, USA), according to the manufacturer’s instructions. The primary antibodies used in this study are listed in Table S1.

#### 2.1.10. Whole-Cell Lysate and Nuclear Extract Preparation

Whole-cell lysate was prepared by homogenizing kidney cortex with RIPA buffer containing protease and phosphatase inhibitors (Thermo Fisher Scientific, Waltham, MA, USA). The lysate was centrifuged at 11,000× *g* for 10 min at 4 °C and supernatant was collected. Nuclear extract was prepared according to the method published by Lahiri and Ge [47]. Briefly, 50 mg of frozen kidney cortex tissue was homogenized in ice-cold cytoplasmic extraction buffer, incubated for 10 min and centrifuged at 2500× *g* for 5 min at 4 °C. The resulting pellet was suspended in nuclear protein isolation buffer, sonicated in ice bath for 1 min at 15 s interval before incubating on ice for 15 min. The samples were then centrifuged at 11,000× *g* for 5 min at 4 °C and the supernatant was collected as the nuclear extract. Protein quantification of whole-cell and nuclear extract was carried out using Bradford reagent using bovine serum albumin as standard. 

#### 2.1.11. Isolation of Mitochondrial Extract

Mitochondrial extract was isolated according to the method described by Rebeca Acin-Perez et al. [48]. Fifty milligrams of frozen kidney cortex was homogenized in ice-cold mitochondrial assay solution (MAS) buffer containing 70 mM sucrose, 220 mM mannitol, 5 mM KH_2_PO_4_, 5 mM MgCl_2_, 1 mM EGTA, and 2 mM HEPES in a prechilled glass-glass homogenizer with 10–20 strokes and centrifuged at 1000× *g* for 10 min at 4 °C. The resulting supernatant was centrifuged again at 10,000× *g* for 10 min at 4 °C and the pellet was suspended in MAS buffer and protein concentration was determined by Bradford method using BSA as standard. The mitochondrial extract was used in respirometry, protein expression, and enzyme activity assays. 

#### 2.1.12. Immunoblotting

Proteins in whole-cell and mitochondrial extracts were solubilized in Laemmli buffer, resolved by SDS-PAGE, and transferred to PVDF membrane using Trans-Blot^®^ Turbo™ transfer system (Biorad, Hercules, CA, USA). The membranes were stained with Ponceau S total protein stain before blocking with 5% nonfat dry milk and incubated with primary antibodies at 4 °C overnight followed by corresponding horseradish peroxidase conjugated secondary antibodies at room temperature for one hour. The band densities were quantified and normalized to total protein using ImageLab software (Biorad, Hercules, CA, USA). The antibodies used in this study are listed in Table S1.

#### 2.1.13. Jess Analysis of Protein Expression

NRF2 protein expression in whole-cell and nuclear extracts was analyzed using specific antibody for NRF2 (Cat# 16396-1-AP, Table S1) in an automated Jess SimpleWestern system (ProteinSimple, San Jose, CA, USA). Briefly, protein extract (0.4 mg/mL) along with other kit components were loaded into the plate according to the manufacturer’s instructions. Protein expression, normalized to total protein stain, was analyzed by integrated Compass SW software of JESS machine.

#### 2.1.14. mRNA Expression Studies by Real-Time Polymerase Chain Reaction

RNA was extracted from the kidney cortex using an RNeasy mini kit (Cat# 74104, Qiagen, Hilden, Germany) and 1 µg of RNA was reverse transcribed into cDNA using RT2 First Strand Kit (Cat# 330404, Qiagen). Polymerase chain reaction was carried out using TaqMan primers specific to transcription factor A, mitochondrial (*Tfam*) (Cat# 4331182, Assay ID Rn00580051_m1, Applied Biosystems, Waltham, MA, USA) and *Nrf2* (Ref: 260550445, Integrated DNA Technologies, Coralville, IA, USA) (*Nrf2*-F 5′-CAGTGGATCTGTCAGCTACTC-3′, *Nrf2*-R 5′-AAGCGACTCATGGTCATCTAC-3′). The results were normalized to 18S rRNA amplified using eukaryotic 18S rRNA endogenous control primer (Cat# 4333760T, Applied Biosystems).

#### 2.1.15. Respirometry in Frozen Mitochondrial Sample

Respirometry in mitochondrial sample derived from frozen kidney cortex was assessed using Seahorse XFe96 analyzer (Agilent, Santa Clara, CA, USA) according to the method of Rebeca Acin-Perez et al. [48]. Briefly, 2 µg of mitochondrial extract with 20 µL MAS buffer was loaded in the Seahorse 96-well plate. The plate was centrifuged at 2000× *g* for 5 min and 115 µL of MAS buffer was carefully added to the wells without disturbing the sample. The sensor cartridge and the plate were loaded and respirometry was carried out according to inbuilt protocol of the instrument. Complex I and complex IV specific oxygen consumption rate was measured using NADH and TMPD/Ascorbate, respectively, as electron donors. The sensor cartridge was loaded with solutions in the order shown in Table S2. Oxygen consumption rate in pmol O_2_/min/µg mitochondrial protein was calculated by the integrated Wave software of the analyzer. 

#### 2.1.16. Mitochondrial Enzyme Activity Assays

The enzyme activities of complex II, II+III coupled, and complex V were measured spectroscopically according to the method previously described by Barrientos A, with slight modifications [49]. Complex II activity was measured by following a decrease in absorbance resulting from the reduction of 2,6-dichlorophenolindophenol at 600 nm. Cortical mitochondrial extract (3 µg) was mixed with 100 µL of medium containing 10 mM KH_2_PO_4_ (pH 7.8), 2 mM EDTA, 1 mg/mL BSA, 80 µm 2,6-dichlorophenolindophenol as acceptor, 0.2 mM ATP and 4 µm rotenone in a 96-well plate and incubated with 10 mM succinate for 10 min at 30 °C. The activity was reported as rate of decrease in absorbance at 600 nm (∆A_600nm_) per microgram of protein.

Complex II+III coupled activity was measured by following an increase in absorbance at 550 nm resulting from the reduction of cytochrome c. Cortical mitochondrial extract (3 µg) was mixed with 100 µL of medium containing 10 mM KH_2_PO_4_ (pH 7.8), 2 mM EDTA, 1 mg/mL BSA, 240 µM KCN, 0.2 mM ATP and 4 µm rotenone along with 10 mM succinate and incubated for 10 min at 30 °C. The activity was measured by adding 40 µm oxidized cytochrome c at 30 s intervals for 5 min. Malonate-sensitive inhibition was followed for an additional 3 min. The activity was reported as rate of increase in absorbance at 550 nm (∆A_550nm_) per microgram of protein.

For complex V activity measurement, medium (200 µL) containing 50 mM Tris (pH 8.0), 5 mg/mL BSA, 20 mM MgCl_2_, 50 mM KCl, 15 µM carbonyl cyanide m-chlorophenylhydrazone (CCCP), 10 mM phosphoenolpyruvate (PEP), 5 µM antimycin A, 2.5 mM ATP, 4 units of lactate dehydrogenase and pyruvate kinase, and 1 mM NADH was incubated at 37 °C for 5 min. Cortical mitochondrial extract (3 µg) was mixed with 20 µL distilled water in a microplate and incubated at 37 °C for 30 s in the spectrophotometer’s chamber. The reaction was initiated by adding the medium to the mitochondrial sample. The decrease in absorbance caused by the reduction of NADH was monitored for 3 min at 340 nm. The activity was reported as rate of decrease in absorbance at 340 nm (∆A_340nm_) per microgram of protein.

Citrate synthase activity was measured in the mitochondrial extract (3 µg) using reaction medium containing 10 mM Tris–HCl, pH 7.5, 0.1 mM DTNB (5,5′-dithiobis (2-nitrobenzoic acid), 0.2 mM Acetyl-CoA and 0.2% Triton X-100. The mitochondrial sample was added to the mixture in the wells, followed by incubation for 5 min at 30 °C. Reaction was initiated by the addition of 0.5 mM oxaloacetic acid and the absorbance was measured at 412 nm, following the reduction of DTNB [50].

### 2.2. Statistical Analysis

The results are presented as mean ± SEM. The differences among the groups were analyzed by one-way ANOVA followed by post-hoc Newman–Keuls test using Graph Pad Prism statistical software (GraphPad ver. 8, San Diego, CA, USA). *p* < 0.05 was considered statistically significant.

## 3. Results

### 3.1. Sulforaphane Treatment Improved NRF2 Signaling in Kidneys of Aged Rats by Increasing Cortical NRF2 Expression, NRF2 Activity, and Decreasing KEAP1 Protein Expression

**Figure 1:**
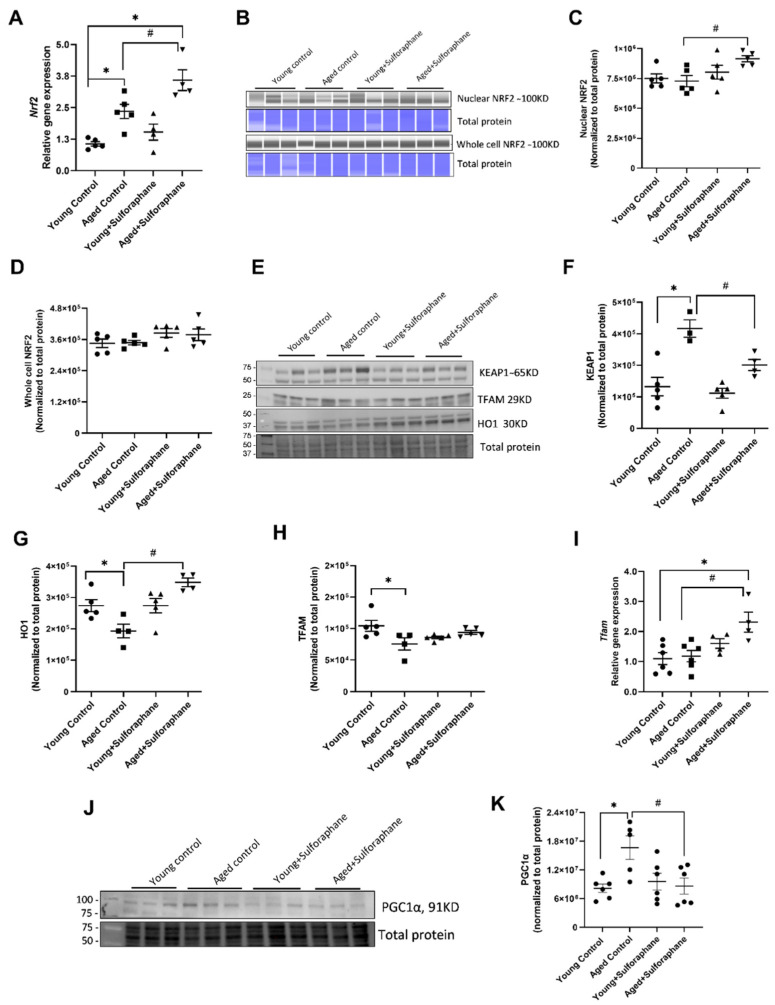
Sulforaphane treatment increases renal cortical NRF2 expression and decreases KEAP1 protein expression in aged rats. (**A**) Kidney cortical *Nrf2* mRNA expression, data normalized to 18 s RNA. Results are presented as mean ± SEM, *n* = 4–5 rats. (**B**) Nuclear and whole-cell NRF2 Western blot images along with total protein. (**C**,**D**) NRF2 protein measured in the nuclear extract and whole-cell extract of kidney cortical tissue, by Jess analysis, Band densities presented as mean ± SEM, *n* = 3–5 rats. Data normalized to total protein stain. (**E**) Western blot images of KEAP1, cortical transcription factor A, mitochondrial (TFAM), and cortical hemeoxygenase 1 (HO1) along with total protein. (**F**) Cortical KEAP1 protein expression. (**G**) HO1 protein expression. (**H**) TFAM protein expression. Band densities presented as mean ± SEM, *n* = 3–5 rats. Data normalized to total protein. (**I**) *Tfam* gene expression, Data normalized to 18 s RNA, *n* = 3–5 rats. (**J**,**K**) images of cortical PGC1 α Western blot and PGC1α protein expression measured in young control (young F344 rats kept on tap water); aged control (aged F344 rats kept on tap water); young+sulforaphane (sulforaphane,15 mg/kg body wt/day in drinking water); aged+sulforaphane. Band densities presented as mean ± SEM, *n* = 5–6 rats. Data normalized to total protein. * *p* < 0.05 significantly different from young control rats; # *p* < 0.05 significantly different from aged control rats, using 1-way ANOVA followed by Newman–Keuls post-hoc test.

Basal cortical *Nrf2* mRNA expression was significantly higher in aged control rats as compared to young control rats (Figure 1A). Sulforaphane treatment further increased cortical *Nrf2* mRNA expression in aged rats as compared to aged control rats (Figure 1A). No significant difference in cortical whole-cell and nuclear NRF2 protein expression was observed between aged control and young control groups (Figure 1B–D). Sulforaphane treatment significantly increased nuclear NRF2 protein expression (Figure 1C) but not total NRF2 expression in aged rats compared to aged control rats (Figure 1D). Aged rats showed a marked increase in renal cortical KEAP1 protein expression when compared to the young control group, which was significantly decreased by sulforaphane treatment (Figure 1E,F). A significant decrease in hemeoxygenase 1 (HO1) protein expression was observed in aged control rats as compared to young control rats (Figure 1E,G). Sulforaphane significantly increased HO1 expression in aged rats as compared to the aged control group (Figure 1E,G). The cortical mRNA expression of *Tfam* in aged control rats was similar to the young control group. However, sulforaphane treatment significantly increased *Tfam* mRNA in aged rats compared to the aged control group (Figure 1I). The increase in *Tfam* mRNA expression in aged rats was also significantly high when compared to young control rats (Figure 1I). Contrary to mRNA expression, TFAM protein expression was significantly lower in the kidney cortex lysates of aged control rats compared to the young control group. Sulforaphane treatment rescued the decrease in TFAM protein expression in aged rats compared to the aged control group (Figure 1E,H). There was a significant increase in peroxisome proliferator-activated receptor gamma coactivator 1-alpha (PGC1α) protein expression in the kidneys of aged rats when compared to young control rats (Figure 1J,K). Sulforaphane treatment significantly decreased PGC1α expression in aged rats compared to aged control rats (Figure 1J,K). Sulforaphane treatment did not affect *Nrf2* mRNA and protein expression, KEAP1, HO1, TFAM or PGC1α protein expression in young rats (Figure 1A–K). 

### 3.2. Sulforaphane Improved Mitochondrial Respiration in Aged Rats

**Figure 2:**
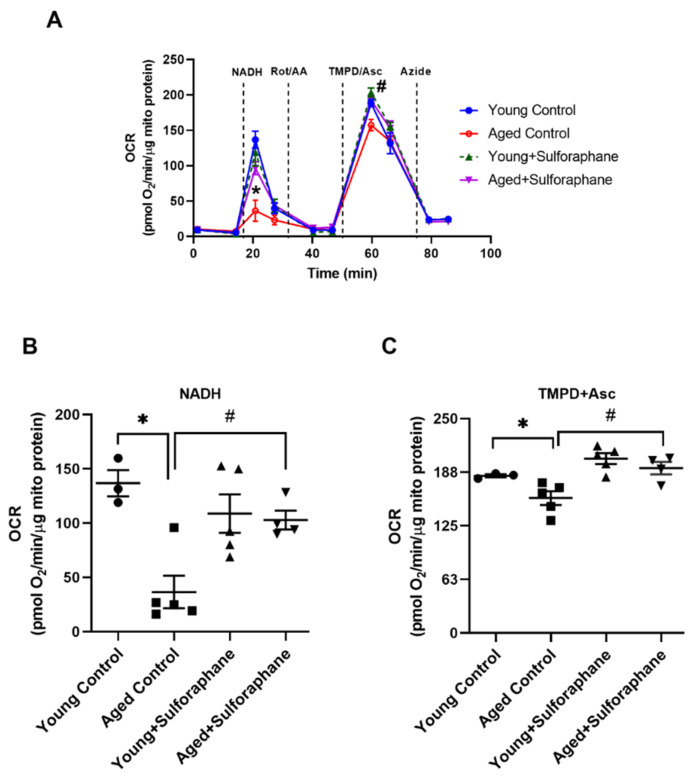
Sulforaphane improves age-mediated decrease in mitochondrial respiration. (**A**) Representative graph showing oxygen consumption rate by renal cortical mitochondria measured upon addition of electron donors specific for complex I (NADH), complex IV (TMPD+ascorbate), and inhibitors for complex I (Rot), complex III (AA), and complex IV (Azide). The vertical lines indicate the timepoint of addition of respective electron donors or inhibitors. (**B**,**C**) Bar graphs showing oxygen consumption rate measured using NADH and TMPD+ascorbate as electron donors, respectively, in the renal cortical mitochondria of young control (young F344 rats kept on tap water); aged control (aged F344 rats kept on tap water); young+sulforaphane (sulforaphane, 15 mg/kg body wt/day in drinking water); aged+sulforaphane. Results are presented as mean ± SEM, *n* = 3–5 rats. * *p* < 0.05 significantly different from young control rats; # *p* < 0.05 significantly different from aged control rats, using 1-way ANOVA followed by Newman–Keuls post-hoc test. Rot, rotenone; AA, antimycin A; TMPD, tetramethyl-p-phenylenediamine.

Mitochondrial respiration using NADH (electron donor specific to complex I) and TMPD+ascorbate (electron donor specific to complex IV) showed significantly decreased oxygen consumption rate (OCR) in the renal cortex of aged control rats compared to young control rats (Figure 2A–C). Sulforaphane significantly increased OCR in aged rats compared to the aged control group (Figure 2A–C). The treatment of young rats with sulforaphane did not change OCR measured using either NADH or TMPD+ascorbate, when compared to young rats (Figure 2A–C).

### 3.3. Effect of Sulforaphane on Electron Transport Chain (ETC) Complex Subunit Expression

**Figure 3:**
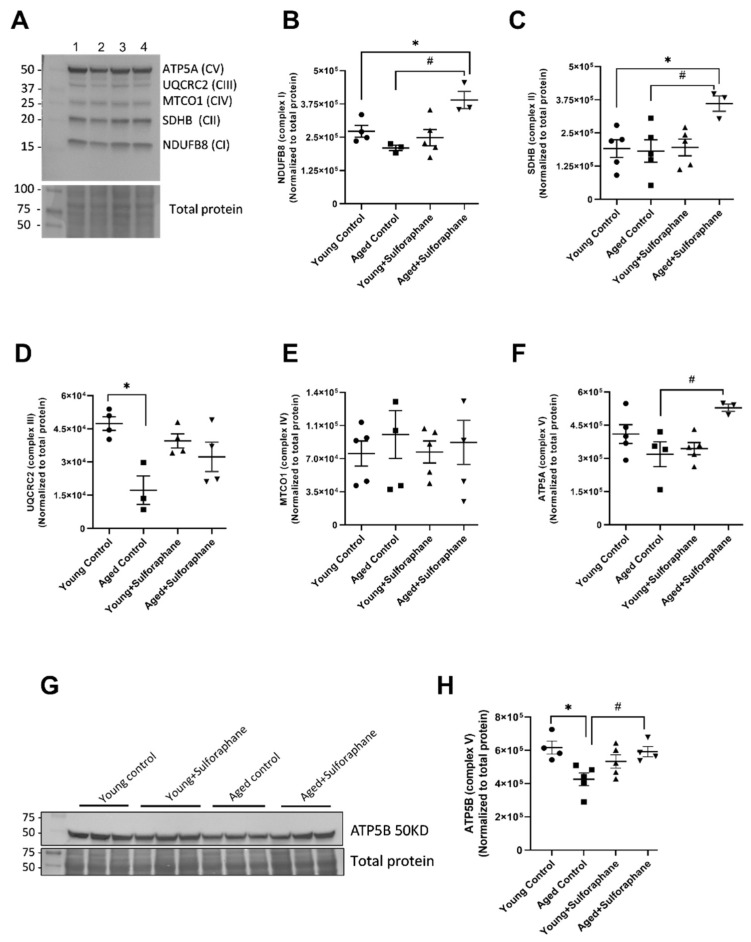
Effect of sulforaphane on the protein expression of ETC complex subunits of mitochondria in the kidney cortex. (**A**) Representative protein blot of complex I to complex V subunits. Lane 1: young control, lane 2: aged control, lane 3: young+sulforaphane, lane 4: aged+sulforaphane. Bar graphs representing protein expression of (**B**) complex I (NDUFB8) subunit, (**C**) complex II (SDHB8) subunit, (**D**) complex III (UQCRC2) subunit, (**E**) complex IV (MTCO1), and (**F**) complex V (ATP5A) subunit. (**G**,**H**) Western blot image of ATP5B and bar graph representing protein expression of ATP5B (complex V subunit) in young control (young F344 rats kept on tap water); aged control (aged F344 rats kept on tap water); young+sulforaphane (sulforaphane (15 mg/kg body wt/day) in drinking water); aged+sulforaphane. Band intensities presented as mean ± SEM, *n* = 3–5 rats. Data normalized to total protein. * *p* < 0.05 significantly different from young control rats; # *p* < 0.05 significantly different from aged control rats, using 1-way ANOVA followed by Newman–Keuls post-hoc test.

Immunoblotting of ETC complexes in cortical mitochondrial extract revealed a non-significant decrease in complex I subunit (NDUFB8), no change in complex II subunit (SDHB), significant decrease in complex III subunit (UQCRC2), no significant change in complex IV subunit (MTCO1), significant decrease in complex V subunits ATP5B and a slight decrease in ATP5A in aged rats in comparison to young control rats (Figure 3A–H). Sulforaphane treatment significantly increased complex I, II, and V subunits (Figure 3A–C,E,G,H) where as it did not affect complex III and IV subunit expression in aged rats when compared to aged control rats (Figure 3D,E). Sulforaphane did not impact the expression of any of the subunits compared to young control rats (Figure 3A–H).

### 3.4. Sulforaphane Increased Mitochondrial Complex V and Citrate Synthase Enzyme Activity but Had No Effect on Complex II and Complex II+III Coupled Activity

**Figure 4:**
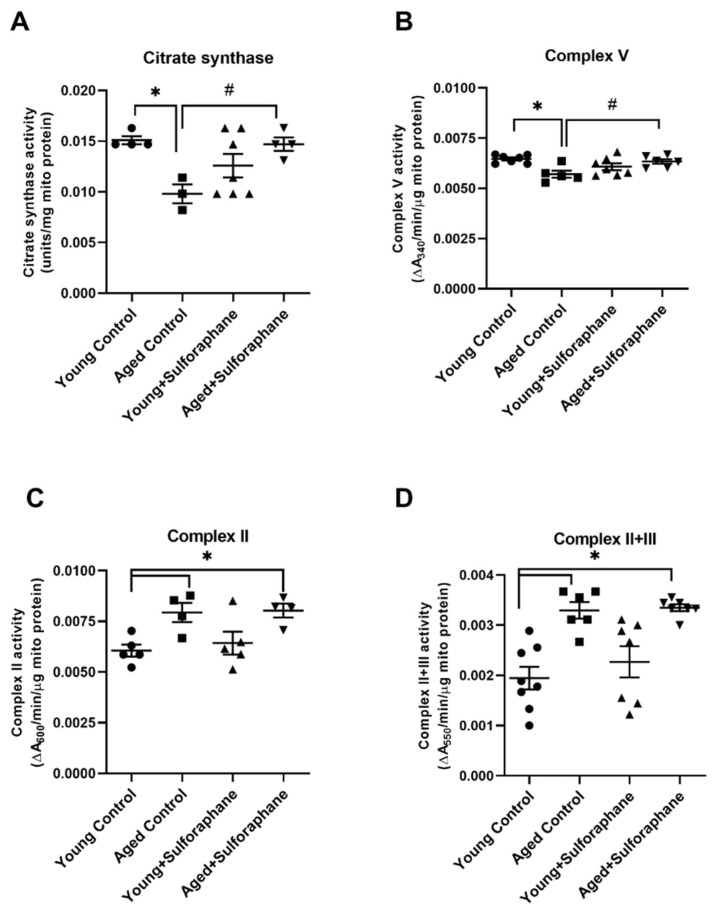
Effect of sulforaphane on mitochondrial enzyme activities. Bar graph representing enzyme activities of (**A**) citrate synthase, (**B**) complex V, (**C**) complex II, and (**D**) complex II+III in young control (young F344 rats kept on tap water); aged control (aged F344 rats kept on tap water); young+sulforaphane (sulforaphane (15 mg/kg body wt/day) in drinking water); aged+sulforaphane. Results are presented as mean ± SEM, *n* = 3–7 rats. * *p* < 0.05 significantly different from young control rats; # *p* < 0.05 significantly different from aged control rats, using 1-way ANOVA followed by Newman–Keuls post-hoc test.

**Figure 5:**
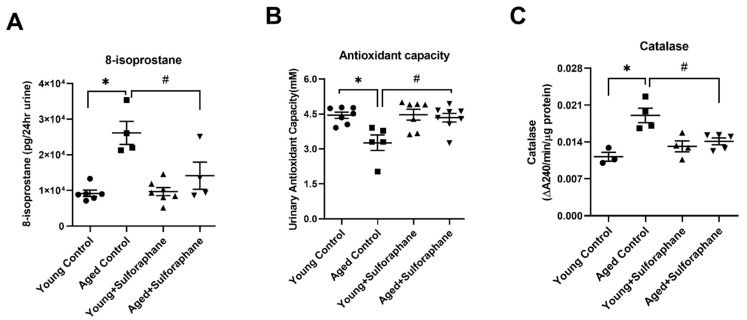
Sulforaphane decreases oxidative stress and improves antioxidant capacity in aged rats. (**A**) 8-isoprostane measured in 24 h urine. (**B**) Total antioxidant capacity measured in bladder urine. (**C**) Catalase activity measured in kidney cortex homogenates of young control (young F344 rats kept on tap water); aged control (aged F344 rats kept on tap water); young+sulforaphane (sulforaphane (15 mg/kg body wt/day) in drinking water); aged+sulforaphane. Results are presented as mean ± SEM, *n* = 3–5 rats for catalase, *n* = 3–6 rats for 8-isoprostane, and *n* = 4–6 rats for antioxidant capacity. * *p* < 0.05 significantly different from young control rats; # *p* < 0.05 significantly different from aged control rats, using 1-way ANOVA followed by Newman–Keuls post-hoc test.

Cortical mitochondrial citrate synthase activity was significantly lower in the kidneys of aged control rats as compared to young control rats (Figure 4A). Sulforaphane treatment in aged rats significantly increased citrate synthase activity when compared to aged control rats (Figure 5A). Complex V activity was also significantly lower in aged rats compared to the young control group (Figure 4B). Sulforaphane significantly increased complex V activity in aged rats when compared to the aged control group (Figure 4B). The results of the complex II and complex II+III coupled assay show significantly high activity in aged control rats as compared to young control rats (Figure 4C,D). Sulforaphane treatment did not change the activity of these enzymes in aged rats as compared to the aged control group (Figure 4C,D). Young rats treated with sulforaphane did not show any difference in citrate synthase, complex II, II+III, and V activity when compared to the young control group (Figure 4A–D).

### 3.5. Sulforaphane Decreased Oxidative Stress by Increasing the Antioxidant Potential in Aged Rat Kidneys

As shown in Figure 5A,B, urinary 8-isoprostane levels, a marker of oxidative stress, were significantly high and total antioxidant capacity was significantly low in aged control rats when compared to young control rats. Sulforaphane treatment significantly reduced urinary 8-isoprostane levels and improved antioxidant capacity in aged rats compared to the aged control group (Figure 5A,B). Catalase activity in the kidney cortex of aged rats was significantly higher when compared to young rats and sulforaphane normalized it in aged rats when compared to the aged control group (Figure 5C). Sulforaphane-treated young rats did not show a change in 8-isoprostane levels, antioxidant capacity, or catalase activity, as compared to young control rats (Figure 5A–C).

### 3.6. Sulforaphane Treatment Reduced Glomerular Damage, Tubualr Injury, and Renal Fibrosis in the Kidneys of Aged Rats

**Figure 6:**
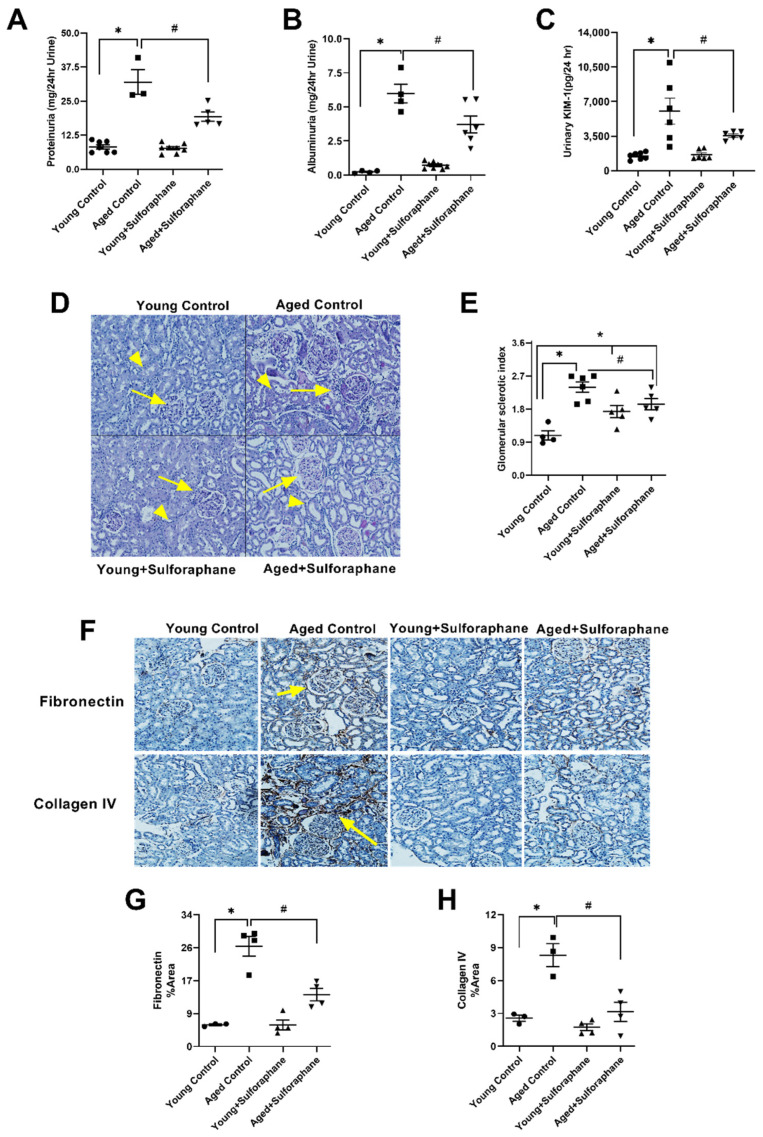
Sulforaphane improves the age-mediated decline in renal function by decreasing the glomerular damage, renal tubular injury and renal fibrosis in kidneys of aged rats. (**A**) Proteinuria. (**B**) Albuminuria. (**C**) Urinary KIM-1 levels. (**D**) Representative images of PAS-stained sections showing glomeruli (arrows) and proximal tubules (arrow heads) in the kidney cortex. (**E**) Bar graph showing glomerular sclerotic index. (**F**) Representative images of fibronectin (top) and collagen IV (bottom) staining (arrows pointing to areas of fibronectin and collagen IV deposition, brown color in glomeruli and interstitial spaces) (**G**,**H**) Bar graph showing % area of fibronectin and collagen IV staining in young control (young F344 rats kept on tap water); aged control (aged F344 rats kept on tap water); young+sulforaphane (sulforaphane (15 mg/kg body wt/day) in drinking water); aged+sulforaphane. Results are presented as mean ± SEM, *n* = 3–7 rats. * *p* < 0.05 significantly different from young control rats; # *p* < 0.05 significantly different from aged control rats using 1-way ANOVA followed by Newman–Keuls post-hoc test.

As illustrated in Figure 6A,B, proteinuria and albuminuria were significantly higher in aged rats when compared to young control rats. Sulforaphane treatment of aged rats significantly decreased proteinuria and albuminuria when compared to aged control rats; however, these parameters remained significantly elevated when compared to young control rats (Figure 6A,B). Treatment of young rats did not cause any change in either of the parameters in comparison to young control rats (Figure 6A,B). Urinary KIM-1, a marker of renal tubular injury, was significantly high in the aged control group as compared to young control rats (Figure 6C), which was significantly decreased by sulforaphane treatment in aged rats when compared to the aged control group (Figure 6C). Sulforaphane treatment did not change urinary KIM-1 levels in young rats when compared to the young control group (Figure 6C). Glomerular sclerotic index, a measure of sclerotic glomeruli, was significantly high in the control and treated group of aged rats when compared to young control rats (Figure 6D,E). As shown in Figure 1D, the glomeruli of the aged control group had increased mesangial matrix expansion, thickening of parietal epithelium of Bowman’s capsule, and disruption of capillary tuft integrity indicating glomerular damage. The presence of protein casts was also observed in surrounding tubules. However, a significant decrease in glomerular damage was observed in aged rats upon sulforaphane treatment when compared to aged control rats, evident from the significantly lesser degree of Bowman’s capsule thickening and mesangial matrix expansion (Figure 6D,E). Surprisingly, when compared to young control rats, the glomerular sclerotic index of young treated rats was significantly high (Figure 6D,E). Expression of renal fibrosis markers, fibronectin and collagen IV showed a marked elevation in aged control rats when compared to the young control group (Figure 6F–H). Treatment of aged rats with sulforaphane caused a significant decrease in the expression of both fibronectin and collagen IV (Figure 6F–H). Kidney sections of young treated rats did not show any difference in expression of renal injury markers when compared to young control rats (Figure 6F–H).

### 3.7. Effect of Sulforaphane on Biochemical Parameters, Blood Pressure and Kidney Weight

Plasma creatinine was significantly higher in in aged control rats compared to young control rats and sulforaphane treatment significantly decreased plasma creatinine in aged rats when compared to aged control rats (Table 1). As indicated in Table 1, the absolute kidney weight of both aged control and aged treated rats were significantly high when compared to the young control group. However, no significant difference was observed in kidney weight/body weight ratio among all the groups. Similarly, no age-mediated change in blood pressure was observed in aged rats, and sulforaphane treatment did not affect the blood pressure of either young or aged rats (Table 1). Fasting blood glucose in aged rats was not significantly different from young control rats (Table 1). However, sulforaphane treatment significantly increased blood glucose levels in aged rats compared to aged controls (Table 1). Sulforaphane treatment of young rats did not have any effect on plasma creatinine and blood glucose when compared to the young control group (Table 1).

Creatinine levels were measured in plasma samples as described in the Methods section. Absolute and relative kidney weights were estimated by weighing the isolated right kidneys. Blood pressure was measured using a pressure catheter transducer as described in the Methods section. Fasting blood glucose was measured after an overnight fast using a digital glucometer in young control (young F344 rats kept on tap water); aged control (aged F344 rats kept on tap water); young+sulforaphane (sulforaphane (15 mg/kg body wt/day) in drinking water); aged+sulforaphane. The results are presented as mean ± SEM, *n* = 4–6 rats. * *p* < 0.05 significantly different from young control rats; # *p* < 0.05 significantly different from aged control rats using 1-way ANOVA followed by Newman–Keuls post-hoc test.

### 3.8. Sulforaphane Decreased Body Weight Gain in Young and Aged Rats but Had No Effect on Daily Food and Water Consumption

**Figure 7:**
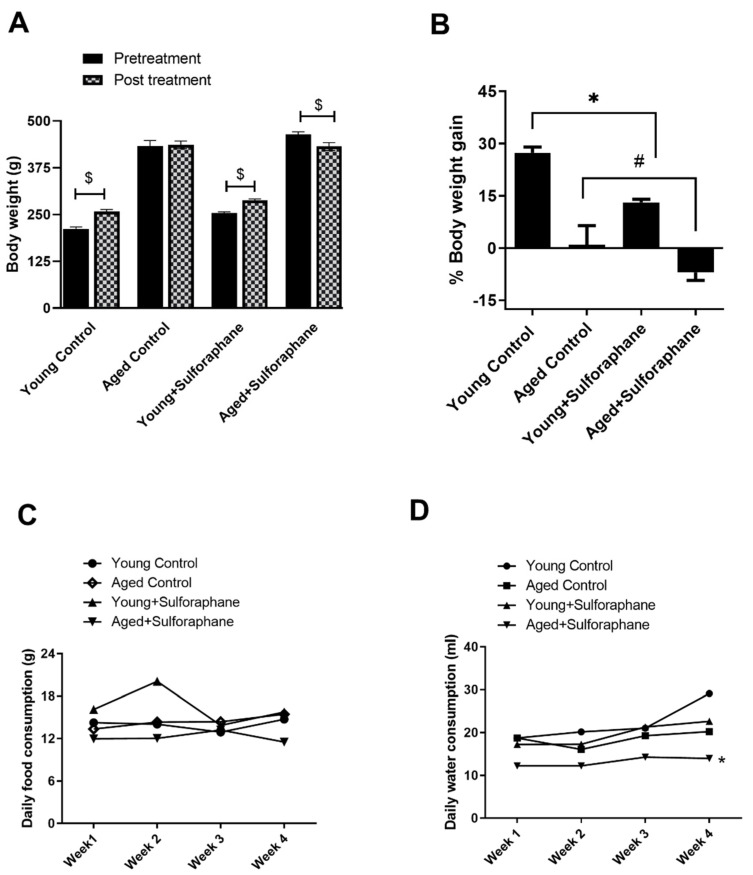
Effect of sulforaphane on body weight gain, food, and water consumption. (**A**) Pre- and post-treatment body weight. (**B**) Percentage body weight gain. (**C**,**D**) Food and water consumption measured weekly in young control (young F344 rats kept on tap water); aged control (aged F344 rats kept on tap water); young+sulforaphane (sulforaphane, 15 mg/kg body wt/day in drinking water); aged+sulforaphane. Results are presented as mean ± SEM, *n* = 5 rats. * *p* < 0.05 significantly different from young control rats; # *p* < 0.05 significantly different from aged control rats; $ *p* < 0.05 vs. pretreatment body weight, using 1-way ANOVA followed by Newman–Keuls post-hoc test.

The post-treatment body weight of both young control and young treated rats was significantly higher than the pretreatment body weights of respective groups (Figure 7A). However, % body weight gain of young rats treated with sulforaphane (13.13%) was significantly lower when compared to % body weight gain of young control rats (27.36%) (Figure 7B). There was no change in the post-treatment body weight of aged control rats when compared to their pretreatment bodyweight; however, sulforaphane caused a significant decrease in the post-treatment body weight of aged rats when compared to their pretreatment body weights (Figure 7A). The daily food consumption of all the groups of rats was similar (Figure 7C). The daily water consumption of aged rats was not significantly different when compared to young control rats, except at week 4 (Figure 7D). Sulforaphane treatment had no effect on the food and water consumption of rats when compared to their age-matched controls (Figure 7C,D).

## 4. Discussion

The present study demonstrates that sulforaphane can rescue age-related impairment in mitochondrial function and renal injury and decrease oxidative stress by activating NRF2 signaling. Aged rats showed significantly elevated oxidative stress, glomerular morphological changes, tubular injury, and renal fibrosis along with marked impairment in mitochondrial respiration, which were reversed by sulforaphane. More importantly, sulforaphane treatment improved NRF2 transcriptional activity by increasing *Nrf2* mRNA expression, promoting nuclear translocation of NRF2, and decreasing KEAP1 expression in aged rats. In young rats, sulforaphane did not impact oxidative stress, mitochondrial respiration, and renal structure when compared to young control rats.

It is well established that oxidative stress plays a vital role in the pathophysiology of age-related kidney diseases [5,6,7,10,11]. In the present study, we also found markedly increased oxidative stress in aged rats. The treatment of aged rats with sulforaphane imparted a beneficial effect in reducing oxidative stress, as evidenced by decreased urinary 8-isoprostane levels and increased antioxidant capacity. Mechanistically, sulforaphane decreased oxidative stress in aged rats by activating the NRF2 signaling pathway. Our results show deficiency in NRF2 stress response in kidneys of aged rats, particularly at the protein level and activity but not at the level of *Nrf2* gene transcription. This was evidenced by significantly increased cortical *Nrf2* mRNA without a corresponding increase in nuclear NRF2 protein expression despite elevated oxidative stress in aged rats. We also observed impaired renal NRF2 activity and reduction in target gene expression in aged rats, a finding similar to a rat model of progressive chronic kidney disease [51]. Several studies have reported alterations in NRF2 activity in both older humans and experimental aging models suggesting a decline in antioxidant potential with advancing age [8,22,23,29]. In the present study, sulforaphane treatment markedly increased renal cortical *Nrf2* mRNA expression, increased NRF2 nuclear protein expression, and improved NRF2 target gene expression, indicating the reversal of impaired NRF2 activity in aged rats. 

NRF2 protein stability is primarily regulated by KEAP1/CUL 3 ubiquitin ligase pathway [52,53], although other pathways, such as β-transducin repeat-containing protein/Cullin1/RING box protein-1 and E3 ubiquitin ligase synoviolin/HRD1, also exist [54]. Our data show significantly elevated KEAP1 protein in aged rat kidneys, which was markedly decreased by sulforaphane. These data are consistent with findings of increased KEAP1 protein in a study of chronic renal failure in 5/6 nephrectomized rats characterized by elevated oxidative stress [38,55]. To our knowledge, this is the first study to show decreased KEAP1 protein expression in response to sulforaphane in aging kidneys. Although the mechanisms of decrease in KEAP1 are not elucidated in our study, sulforaphane could have a role on *Keap1* promoter methylation. A study in the cellular model of Alzheimer’s disease showed that sulforaphane increased *Nrf2* expression by decreasing *Nrf2* promoter demethylation, so its role on *Keap1* promoter is also a possibility [56]. Taken together, our results demonstrate that decreased NRF2 activity and elevated KEAP1 contributed to impaired NRF2 signaling disrupting redox homeostasis in the aged rat kidneys. Sulforaphane treatment increased NRF2 signaling in aged rats and thereby improved the antioxidant defense. Furthermore, the absence of NRF2 activation by sulforaphane in young rats suggests a selective effect of sulforaphane on NRF2 signaling during increased oxidative stress. Since redox homeostasis is normal in the kidneys of young rats, excessive NRF2 activation beyond basal levels might be a futile process or it could be a regulatory measure to prevent deleterious effects of overt NRF2 activation in young rats. An uncontrolled upregulation of NRF2 is associated with tumorigenesis and drug resistance in various cancers [57,58]. It could also be conferred that multiple cellular regulatory pathways might exist to prevent the persistent activation of NRF2 under normal conditions. KEAP1 is the widely studied regulator that keeps the basal NRF2 levels in check. Alternate pathways such as transcriptional regulation, post-transcriptional regulation, and KEAP1 independent regulators such as β-transducin repeat-containing protein/Cullin1/RING box protein-1 and E3 ubiquitin ligase synoviolin/HRD1 might play a role in preventing further activation of NRF2 in normal conditions [21,54]. 

Impaired mitochondrial biogenesis and function is a hallmark of aging in both humans and rodents and is reported in many pathologies, including kidney diseases [12,59,60]. Based on the regulatory role of NRF2 on mitochondria [61], it can be hypothesized that impaired NRF2 activity during aging can lead to compromised renal mitochondrial function and can be rescued by NRF2 activation. The effect of NRF2 on mitochondrial biogenesis in aging kidneys was assessed by measurement of TFAM and PGC1α expression. TFAM is a nuclear-encoded transcription factor of mitochondria that facilitates mitochondrial DNA transcription, enhances its stability, and maintains mitochondrial DNA copy number [62]. We found a significant decrease in TFAM protein expression in aged rats consistent with previous reports of decreased TFAM protein in aged mice brains and kidneys, aged rat liver, and kidneys of humans and animals with CKD [63,64,65]. Sulforaphane treatment significantly increased TFAM mRNA and rescued TFAM protein expression in aged rat kidneys compared to aged control rats, suggesting a protective effect of sulforaphane on mitochondrial biogenesis. PGC1α, a transcriptional coactivator, plays a key role in mitochondrial biogenesis, metabolism, and antioxidant defense mechanisms during oxidative stress [66,67,68]. Furthermore, PGC1α potentiates antioxidant response by increasing NRF2 expression [69]. Our data show a significant increase in PGC1α expression in aged rat kidneys, which could be one of the reasons for the increase in *Nrf2* mRNA expression in aged rat kidneys. Sulforaphane significantly decreased PGC1α expression in aged rat kidneys.

Mitochondria supply ATP to meet the cellular demands, through oxidative phosphorylation or respiration carried out by the ETC complexes, complex I to complex IV and ATP synthase (complex V) [70]. Our results show significantly decreased mitochondrial respiration in aged rat kidney cortex, which is in agreement with previous reports of diminished mitochondrial respiration during aging [10,71,72]. NRF2 is known to regulate mitochondrial respiration in a variety of ways, including controlling the availability of substrates and expression of ETC complex subunits [35,36]. Sulforaphane significantly improved mitochondrial respiration in aged rats. A striking finding of our study is elevated complex II and complex II+III coupled enzyme activity. The reason for increased complex II activity is not clear, but two possible reasons can be envisioned for this observation. First, it could be a compensatory measure in response to decreased complex I function. Loss of complex I activity promotes increased electron flux through complex II to complex III [73]. This increased electron flux from complex II overwhelms the ubiquinone (CoQ) pool, resulting in reverse electron transport to complex I and further generation of ROS [74,75]. The second possibility could be a negative impact on the functioning of complex II and complex III due to the disruption of super complexes caused by dysfunctional complex I. ETC complexes exist as super complexes (complex I, complex III, and complex IV), complex I and complex III, or complex III and complex IV [76]. Sulforaphane treatment showed no effect on the elevated complex II and complex II+III assay in aged rats. In agreement with previous reports, our findings also indicate an age-dependent decrease in complex V activity, ATP5A, and ATP5B protein expression in the aged rat kidneys, which was remarkably increased by sulforaphane [77]. Taken together, aged rat kidneys exhibited significantly impaired mitochondrial function evident from decreased mitochondrial respiration, impaired ETC enzyme activities, and citrate synthase activity. Our findings suggest that sulforaphane plays a key role in mitigating age-mediated renal mitochondrial dysfunction. Collectively, our study provides significant evidence that impaired renal NRF2 activity is implicated in mitochondrial dysfunction of aged rats, which can be improved through NRF2 activation by sulforaphane.

Kidney diseases are manifested by structural changes in the glomeruli and extracellular matrix deposition, and renal functional impairment. Herein, our results show that aged rats showed significant decline in renal function, as evidenced by increased plasma creatinine and structural changes such as glomerular sclerosis, collagen, and fibronectin deposition. Compromised NRF2 signaling during aging, in part, could be a causative factor in the development of renal fibrosis. Previous reports showed that NRF2 signaling modulates TGFβ1-mediated transition of epithelial cells to fibroblastic cells in renal tubules by inhibiting SMAD signaling via the enhancement of SMAD7 levels [78]. TGFβ1 is a profibrogenic cytokine implicated in extracellular matrix deposition [78]. SMAD7 forms a complex with TGFβ1 receptor and leads to its proteasomal degradation by recruiting E3 ubiquitin ligase [79]. Sulforaphane reduced the structural anomalies observed in aged rats. Our data are in line with the findings of Pan et al., who showed that NRF2 activation ameliorated renal fibrosis [41] and therefore provide evidence that NRF2 activation during aging can ameliorate renal injury.

## 5. Conclusions

**Figure 8:**
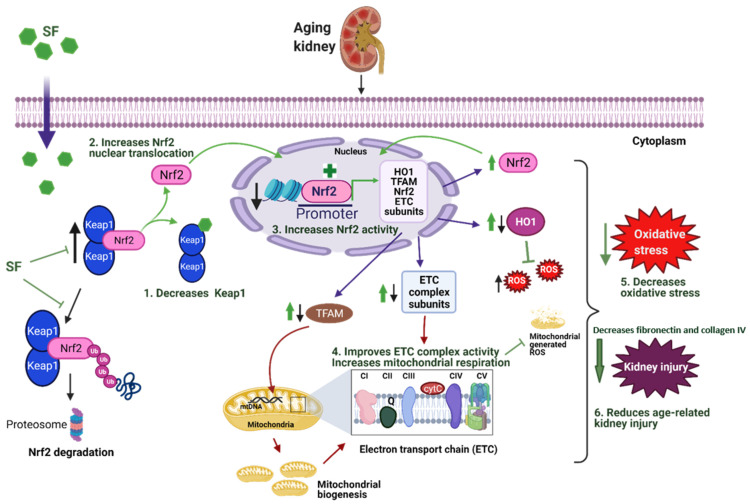
Schematic of NRF2 signaling in the aged kidney and effect of sulforaphane. Black arrows show sequence of pathways in the aging kidney, and green arrows show effect of sulforaphane. NRF2 signaling is impaired in aging kidney due to increased KEAP1, decreased NRF2 nuclear translocation and promoter binding activity. As a result, NRF2 target genes, *Ho1*, *Tfam*, and *ETC* subunit expression is decreased, leading to compromised mitochondrial respiration, increased oxidative stress, and kidney injury. Sulforaphane increases *Nrf2* gene expression, decreases KEAP1 protein, increases NRF2 translocation, and increases NRF2 transcriptional activity, leading to gene expression of *Nrf2* and target genes. Increased NRF2 targets, HO1, TFAM, and ETC subunits improves mitochondrial respiration and subsequently ameliorates oxidative stress and reduces kidney injury by decreasing renal injury markers, fibronectin and collagen IV, during aging. SF, sulforaphane; HO1, heme oxygenase 1; TFAM, transcription factor A, mitochondrial; ETC, electron transport chain; Ub, ubiquitin; ROS, reactive oxygen species. Created with Biorender.

In conclusion, age-related kidney dysfunction is manifested by impairment in mitochondria along with drastically elevated oxidative stress. Our data suggest that sulforaphane can significantly improve renal mitochondrial function and reduce kidney injury through activation of NRF2 signaling in kidneys of aged rats, as shown in Figure 8. This study supports NRF2 as a therapeutic target and highlights the therapeutic potential of sulforaphane in treating age-related kidney disease. Sulforaphane might also confer broader benefits to the elderly who are at a risk of cardiovascular complications that arise due to renal dysfunction.

## 6. Limitations of the Study

Our data highlight the decrease in KEAP1 protein and subsequent increase in NRF2 nuclear translocation in aged rats after sulforaphane treatment; however, the modulation of NRF2 by KEAP1-independent pathways cannot be excluded. It would be interesting to study how these mechanisms influence kidney function during aging. In addition, our data suggest the protective role of the sulforaphane-mediated activation of NRF2 signaling on kidney function during aging. However, to shed light on the cause and effect of sulforaphane’s protective role through NRF2, in aging kidney disease, an NRF2 inhibition strategy should be employed. While our study focused on the potential of sulforaphane to activate the NRF2 signaling pathway, further studies are warranted to explore other molecular mechanisms influenced by sulforaphane, namely, cell growth and proliferation. Moreover, the study only included male animals, and the role of sex as a variable warrants further investigation.

## Figures and Tables

**Table 1:** Effect on sulforaphane on biochemical parameters, blood pressure and kidney weight.

Parameter	Young Control	Aged Control	Young+Sulforaphane	Aged+Sulforaphane
Plasma creatinine (mg/dL)	1.51 ± 0.14	3.03 ± 0.77 *	1.61 ± 0.25	1.33 ± 0.19 #
Absolute kidney weight (mg)	920.00 ± 51.15	1378.40 ± 37.51 *	1000.60 ± 7.98	1351.12 ± 32.02 *
Relative kidney weight (mg/g body wt)	3.19 ± 0.05	3.27 ± 0.03	3.38 ± 0.03	3.41 ± 0.15
Blood pressure (mmHg)	103.62 ± 2.48	101.90 ± 1.74	107.36 ± 0.26	100.57 ± 5.38
Blood glucose (mg/dL)	120.75 ± 2.17	112.00 ± 4.73	121.60 ± 2.73	128.20 ± 2.73 #

* *p* < 0.05 vs. Young control; # *p* < 0.05 vs. Aged Control.

## Data Availability

All data generated for this study are contained within the article and Supplementary Materials.

## Supplementary Materials

The following supporting information can be downloaded at: https://www.mdpi.com/article/10.3390/antiox11010156/s1, Figure S1: Effect of sulforaphane on body weight gain, food, and water consumption, Table S1: List of antibodies used in the study, Table S2: Substrates used in respirometry in frozen kidney samples.
