# Supplementary material for: Age-Related Mitochondrial Impairment and Renal Injury Is Ameliorated by Sulforaphane via Activation of Transcription Factor NRF2
Source: Antioxidants (Basel). 2022 Jan 14;11(1):156. doi: 10.3390/antiox11010156 (PMC8772968; doi:10.3390/antiox11010156)
Supplement: Supplementary file 1 [file antioxidants-11-00156-s001.zip › antioxidants-1517027-supplementary.pdf]

Table S1. List of antibodies used in the study.

S.no	Name of the Antibody	Catalog Number & Source	Dilution	Company
1	NRF2	16396-1-AP, rabbit polyclonal	1:500 (JESS)<br>1:1000 (WB)	ProteinTech
2	KEAP1	sc-365626<br>mouse monoclonal	1:1000 (WB)	Santa Cruz Biotechnology
3	Hemeoxygenase 1	ab13243<br>rabbit polyclonal	1:1000 (WB)	Abcam
4	TFAM	22586-1-AP<br>rabbit polyclonal	1:1000 (WB)	Protein Tech
5	ATP5B	ab14730<br>mouse monoclonal	1:1000 (WB)	Abcam
6	OXPHOS<br>antibody cocktail	MS 604-300<br>mouse monoclonal	1:1000 (WB)	Abcam
7	Collagen IV	ab6586<br>rabbit polyclonal	1:200 (IHC)	Abcam
8	Fibronectin	ab2413<br>rabbit polyclonal	1:200 (IHC)	Abcam

9	PGC1α	ab191838<br><br>rabbit polyclonal	1:500 (WB)	Abcam
10	Anti-rabbit HRP<br><br>secondary antibody	7074S	1:5000 (WB)	Cell signaling
11	Anti-mouse HRP<br><br>secondary antibody	Sc-2005	1:5000 (WB)	Santa Cruz<br><br>Biotechnology

Table S2. Substrates used in respirometry in frozen kidney samples.

**Port**	**Substrate/Inhibitor**	**Final Concentration in the Well (1X)**	**Concentration Loaded in the Port (10X)**
**A**	NADH	1 mM	10 mM
**B**	Rotenone (Rot)<br>+<br>Antimycin A (AA)	2 µM (Rot)<br>+<br>4 µM (AA)	20 µM (Rot)<br>+<br>40 µM (AA)
**C**	TMPD + Ascorbate	0.5 mM TMPD + 1 mm Ascorbate	5 mM TMPD in<br>10 mM Ascorbate
**D**	Sodium Azide	50 mM	500 mM

Oxygen consumption rate was measured in renal cortical mitochondria as described in Methods section by addition of electron donors specific for complex I (NADH), complex IV (TMPD+ascorbate), and inhibitors for complex I (Rot), complex III (AA), and complex IV (Azide) to the injection port of assay plate of Agilent Seahorse XFe96 analyzer. Rot, rotenone; AA, antimycin A; TMPD, tetramethyl-p-phenylenediamine.
